# Genetic Identification and Risk Factor Analysis of Asymptomatic Bacterial Colonization on Cardiovascular Implantable Electronic Devices

**DOI:** 10.1155/2014/725163

**Published:** 2014-11-02

**Authors:** Xian-Ming Chu, Bing Li, Yi An, Xue-Bin Li, Ji-Hong Guo

**Affiliations:** ^1^Department of Cardiology, The Affiliated Hospital of Qingdao University, Qingdao 266100, China; ^2^Department of Biology, Medical College of Qingdao University, Qingdao 266021, China; ^3^Department of Cardiac Electrophysiology, Peking University People's Hospital, Beijing 100044, China

## Abstract

Asymptomatic bacterial colonization of cardiovascular implantable electronic devices (CIEDs) is widespread and increases the risk of clinical CIED infection. The aim of the study was to evaluate the incidence of bacterial colonization of generator pockets in patients without signs of infection and to analyze the relationship with clinical infection and risk factors. From June 2011 to December 2012, 78 patients underwent CIED replacement or upgrade. Exclusion criteria included a clinical diagnosis of CIED infection, bacteremia, or infective endocarditis. All patients were examined for evidence of bacterial 16S rDNA on the device and in the surrounding tissues. Infection cases were recorded during follow-up. The bacterial-positive rate was 38.5% (30 cases); the coagulase-negative *Staphylococcus* detection rate was the highest (9 cases, 11.5%). Positive bacterial DNA results were obtained from pocket tissue in 23.1% of patients (18 cases), and bacterial DNA was detected on the device in 29.5% of patients (23 cases). During follow-up (median 24.6 months), two patients (6.7%, 2/30) became symptomatic with the same species of microorganism, *S. aureus* and *S. epidermidis*. Multivariable logistic regression analysis found that the history of bacterial infection, use of antibiotics, application of antiplatelet drugs, replacement frequency, and renal insufficiency were independent risk factors for asymptomatic bacterial colonization.

## 1. Introduction

Cardiovascular implantable electronic devices (CIEDs) have been used since 1950s and have helped save the lives of many patients. A recent survey involving 61 countries indicated that in 2009 the number of implanted pacemakers was 1,002,664 with 264,824 being replaced and the number of implanted cardioverter-defibrillators was 328,027 with 105,620 being replaced [1]. However, consequent CIED-related infection has become a difficult problem which is difficult to diagnose and treat and expensive and is associated with a high fatality rate. In addition, the removal of CIEDs and electrode wires is a high-risk operation. Recently it has been reported that the occurrence rate of CIED infection was 1~7% [2–4]. To effectively control CIED infection, it is necessary to discover the risk factors for CIED infection and to provide a specific prevention strategy.

Bacterial biofilms and bacterial colonization on the surface of implanted devices* in vivo* might lead to clinical infection [5–8]. Recent research revealed that asymptomatic bacterial colonization on CIEDs might be ubiquitous and increase the risk of clinical CIED infection [9–12]. Early diagnosis of patients with asymptomatic bacterial colonization is an important basis to apply specific preventative measures and to reduce clinical CIED infection. In the present study, bacterial identification based on the 16S rRNA gene was carried out to study the bacteria in pocket tissues and on the surface of the impulse generator in patients with replacement of CIEDs. The relationship between related risk factors of bacterial colonization and clinical CIED infection was also analyzed.

## 2. Methods

### 2.1. Patients

A total of 78 patients who had replaced or upgraded CIEDs between June 2011 and December 2012 were enrolled consecutively. Patients who were clinically diagnosed with CIED infection, including pocket infection, bacteremia, and infective endocarditis, were excluded. Clinical characteristics and laboratory examination results were collected. The prospective registration and follow-up were carried out. Based on the Declaration of Helsinki, all patients signed medical informed consent forms to participate in this study, and the study was approved by the Ethics Committee of the Affiliated Hospital of Qingdao University.

### 2.2. Collection of Clinical Characteristics

The following characteristics were collected: age, gender, body mass index, reason of replacing or implanting the CIED, date of implantation, frequency of replacement, usage of temporary pacemaker, and type of the pacemaker. Past medical history included coronary heart disease, hypertension, atrial fibrillation, diabetes, renal insufficiency, chronic systolic heart failure, and chronic obstructive pulmonary disease (COPD). Bacterial infection history in the past five years contained upper respiratory infection, lower respiratory infection, urinary system infection, soft tissue infection, digestive system infection, and infection in other parts. The history of surgery in the past five years that required hospitalization was also recorded. Medication history was composed of immunosuppressive agents, anticoagulant drugs (warfarin), antiplatelet drugs (aspirin or clopidogrel), intravenous antibiotics, and oral antibiotics. Laboratory examinations consisted of ejection fraction, white blood cell count, C reactive protein, hemoglobin, total serum protein, and albumin.

The comorbidities included diabetes, renal insufficiency (glomerular filtration rate <60 mL/min × 1.72 m^−2^), systolic heart failure (NYHA ≥ II class, ejection fraction <45%), and chronic heart disease (diagnosed coronary heart disease, NYHA classes III and IV, or hypertension that need to be treated by ≥3 drugs). Antibiotic therapy was defined as any sequential oral or intravenous antibiotic therapy more than seven days in the past five years.

### 2.3. Collection of Specimens

During the replacing operation, 0.5 g of the pocket tissue was sampled and biofilms on the surface of the CIED were collected using a sterile scalpel. All the specimens were reserved in sterile containers and immediately preserved at −80°C.

### 2.4. Bacterial Genetic Determination [13]

Pocket tissues and the samples obtained from generators surface were washed with phosphate buffer solution (PBS) and genomic DNA was extracted using Wizard genomic DNA extraction kit (Promega, USA) according to the manufacturer's protocol.

In order to accurately determine the bacteria in the sample, universal primers (upstream primer: AGAGTTTGATCCTGGCTCAG; downstream primer: AGTAAGGAGGTGATCCAACCGCA) were designed to target the conserved region of the 16S rRNA gene (rDNA) according to* Escherichia coli* (GenBank J01695), which could amplify nearly all bacteria by PCR (7700, Perkin Elmer, USA). The positive band indicated the presence of bacteria in the sample. The PCR product was purified using Wizard PCR Preps DNA Purification System (Promega) and then ligated into the pGEM-T Easy Vector (Promega). The ligation product was transformed into the* E. coli* strain JM109. Colonies containing the inserted 16S rRNA gene inserts were identified using blue/white screening. Plasmid DNA from candidate colonies was extracted and restricted with* Eco*RI. The inserted 16S rRNA gene sequence was then sequenced and identified by the BLAST algorithm against EMBL and GenBank databases.

### 2.5. Clinical Procedure

Routine checks included a chest X-ray and a cardiac color ultrasound. Before the operation, routine blood tests were carried out. The first generation of cephalosporin antibiotics was injected once before operation and persisted for 72 h after the operation. Patients were subjected to a chest X-ray, wound check, and routine pacemaker program control follow-up before leaving the hospital one week after the operation. Routine follow-up was carried out for all patients every three months after the operation (16–34 months).

### 2.6. Criteria of CIED Infection during Follow-Up

Clinical symptoms included local inflammation in the pocket tissue, such as erythema, fever, fluctuation, wound dehiscence, decay, tenderness, and suppuration. The diagnosis of infective endocarditis was in accordance with the European Society of Cardiology (ESC) criteria [14]. Verified CIED infection by the same microbes was based on the identification of microbes in the operation and the culture result after infection.

### 2.7. Statistical Analysis

Normally distributed continuous variables were expressed as means ± SD and continuous variables of skewed distribution were expressed as median values. Comparison between two continuous variables of normal distribution was carried out using the *t*-test. Comparison of classified variables was performed by the chi-square test or Fisher's exact test. Correlation between clinical characteristics and CIED asymptomatic bacterial colonization was analyzed by the multivariate logistic regression analysis. The software used for statistical analysis was SPSS18.0.

## 3. Results

### 3.1. PCR Amplification Results of the 16S rRNA Gene from Partial Patients

**Figure 1:**
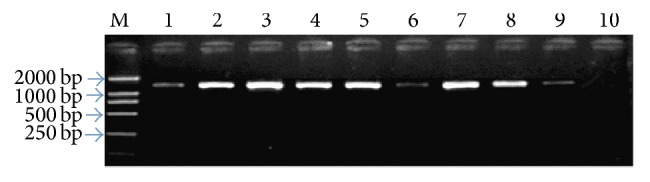
UP-PCR results of some patients. We have detected all the patients and finally we chose some positive bacteria and carried out the electrophoresis, so every line had PCR products. The deeper the stripe, the more the bacteria. Lines 1–9 represented PCR product of the 16S rRNA gene of* Staphylococcus aureus*,* Staphylococcus epidermidis*,* Pseudomonas aeruginosa*,* Escherichia coli*,* Streptococcus viridans*,* Staphylococcus saprophyticus, Corynebacterium parvum*,* Klebsiella pneumoniae*, and* Enterobacter cloacae*; M for DL2000 DNA Marker; ten for negative control.

The amplified fragment length was 1532 bp just as shown in Figure 1.

### 3.2. Identification Results of Restriction Enzyme Digestion of Recombinant Plasmid

**Figure 2:**
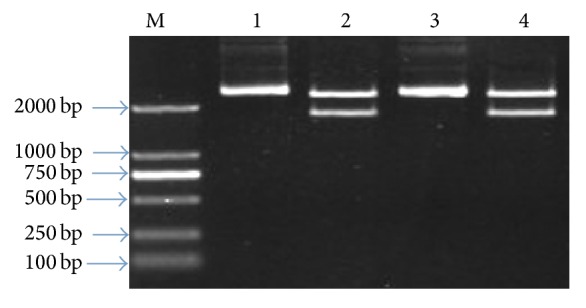
Identification of enzyme digestion of 16S rRNA gene recombinant. 1, 3: enzyme-digested product of 16S rRNA gene recombinant plasmid; 2, 4: blank pGEM-T plasmid; M for DL2000 DNA Marker.

Figure 2 showed the identification results of restriction enzyme digestion of recombinant plasmid.

### 3.3. Identification Results of the Bacterial 16S rRNA Gene Are Shown in Tables 1 and 2

Bacteria were detected in 38.5% of 78 patients, among which 23.1% were found in pocket tissues and 29.5% in biofilms. The percentage of coagulase-negative* Staphylococcus* was the maximum. In total, eleven patients were positive in both pocket tissues and biofilms, of which the bacteria of two patients were inconsistent in pocket tissues and biofilms, one of* E. coli* and* Corynebacterium parvum* and another of* Pseudomonas aeruginosa* and* S. epidermidis*.

### 3.4. Single Factor Analysis of Asymptomatic Bacterial Colonization Risk Factors

See Tables 3, 4, and 5.

### 3.5. Multivariate Logistic Regression Analysis of Asymptomatic Bacterial Colonization Risk Factors

See Table 6.

### 3.6. Clinical Follow-Up

All patients were viable during the follow-up period with no deaths. The median follow-up time was 24.6 months (range, 16–34 months; mean, 25.6 ± 5.8). Two patients (6.7%, 2/30) in the group of positive bacterial detection presented with CIED infection, which occurred 3 and 11 months after the operation, respectively. In the two patients, when CIED was replaced, the bacterial identification results were* S. aureus* and* S. epidermidis*, in line with the cultural results after infection. The patient infected with* S. aureus* had a diagnosis of cancer. Ultrasound confirmed wire vegetations and infective endocarditis with a positive blood culture. The other patient with pocket infection showed red, swollen, and diabrotic symptoms. The result of tissue culture was* S. epidermidis*, but the blood culture was negative.

## 4. Discussion

Although application of CIED is beneficial, it can lead to serious complications such as infection [2–4, 15, 16]. Replacement of CIED or repeated interventional treatments can increase the probability of infection [2–4]. Harcombe et al. have reported that the probability of infection after the replacement of CIED was about five times more than that after the first implantation [17]. Several hypotheses could explain an equilibrium between the human host and bacteria [18]. When the balance is broken, bacteria are destroyed or infection occurs. Many factors may influence this balance, such as the number of bacteria or the addition of a new infection, the virulence of bacteria and their ability to adapt to the unfavourable environment, and the defensive capacity of the host [9–12]. Repeating interventional treatments decreased patients' defense to pathogens [10].

The bacteria causing apparent pocket infection can be cultured and identified. However, as a potential infection, only 20–30% of bacteria could be identified by traditional culture-dependent methods. It is not well understood whether the pathogens are not culturable or if it is an aseptic inflammation [19–22]. Classification of pathogens used to be based on isolate, morphological, biochemical, and immunological methods which are time-consuming, poorly specific, and low sensitive. However, the 16S rRNA gene sequence analysis technology has allowed bacterial evolution to be confirmed by experimental investigation, which has revolutionized in bacterial taxonomic history. The homology of ancient 16S rRNA is high, and the gene contains both conserved and variable sequences. The molecular size of the gene is suitable to operate and the sequence variation adapts to evolutionary distance. Therefore the 16S rRNA gene has been the most common and useful molecular clock in bacterial taxonomy [20, 21].

In the present study, the total positive rate for bacterial determination in 78 asymptomatic patients with CIED replacement reached 38.5%. The major bacterium was coagulase-negative* Staphylococcus* (11.5%). The high positive results were consistent with previous studies and suggested that similar ubiquitous bacterial colonization was present [9, 12, 23]. Research indicated that one-third of implantable cardioverter-defibrillator (ICD) patients were positive for microbial swab culture in pocket tissues and drawn wires when replacing generator and wires [10].

Provided that bacterial DNA was rapidly degraded after death [24, 25], it could be considered that the organism detected was derived from microfunctional groups but not molecular residues from contamination during the last operation. In the present study, DNA was isolated from 29.5% of biofilms on the CIED surface and 23.1% of subcutaneous pocket tissues and 14.1% from both. This result showed that microbes were easy to exist not only on the CIED but also in tissues near the CIED. During the follow-up period, 93.3% (28/30) patients with positive bacteria had no clinical symptoms of infection. This suggested that there were uninjurious microbial communities on the generators surface. But, under some conditions, the balance was destroyed and pathogens (e.g.,* Staphylococcus*) might lead to infection. However, the specific mechanism needed to be further verified. The bacterium growing on the skin, for example,* C. parvum*, was considered to be probably contaminated during sampling.

Previous study has shown that* S. aureus* and* S. epidermidis* that were detected by culture might be clinically related to deadly pathogens that caused instrument infection [2–4, 15, 16]. The present study confirmed this point.* Staphylococcus* were detected in 10 patients (Table 2). During follow-up, two patients (6.7%) who were positive for bacterial determination suffered from CIED infection. The bacteria detected in the CIED replacement were identified as* S. epidermidis* and* S. aureus*, which were in accordance with the culture result after infection and shared the same antimicrobial susceptibility. The two bacteria belonged to the common bacteria in CIED infection in the previous study [2–4, 15, 16]. Therefore bacterial identification and antimicrobial susceptibility test for CIED could guide the correct antibiotic treatment. In order to kill bacteria, new treatment methods were being constantly generated. A recent study revealed that direct current could kill* S. aureus *in biofilms on the surface of CIEDs [26].

There are factors associated with a weakened immune response or predisposition to repetitive bacteraemias, which have been shown to predispose to the infection [27]. The risk factors include renal failure [21, 28], diabetes and congestive heart failure [21], the number of previous operations [21, 22, 29], increasing number of leads [22], experienced bacteraemias [30], and vegetations on the leads [27, 31]. But a previous study showed that common risk factors for device infection did not correlate with the presence of DNA [9]. In the present study, analyses of risk factors related to asymptomatic bacterial colonization indicated that bacterial infection history, antibiotic history, usage of antiplatelet drugs, two replacements of CIED, and renal insufficiency were independent risk factors for asymptomatic bacterial colonization. The history of bacterial infection and antibiotic usage prompted immune dysfunction, and the patients might be repeatedly exposed to bacterial infections or bacteremia, which might increase the pathogenic bacteria migration to the surface of implants in the body. Use of antiplatelet drugs may cause microbleeding in pockets, and renal insufficiency was often associated with immune and circulation dysfunction, which might be susceptible to microbial colonization.

Repetitive operations such as replacement and upgrade of a pacemaker could easily cause infection [3, 10, 32]. The probability of infecting complications significantly increased for the patients who received treatment for a complex implantable device [32, 33]. The infection rate was 5.5% for the young patients that received an average of two pacemaker implanting operations, five times higher than that for the first operation [33]. Before, secondary intervention for hematomas and movement of the wire were the two factors that easily lead to infection, and the odds ratio (OR) reached up to 15.04 [33]. Harcombe et al. revealed that the occurrence rate of complications caused by the replacement of a pacemaker reached 6.5%, while that caused by first implantation of the pacemaker was 1.4% [17]. The complications resulted from the erosion and infection of the implantable device [17]. In total, 80% of patients with clinical infection had received the pacemaker implanting operation on average twice or more than twice, suggesting that repeated implanting significantly increased the probability of infection. The infection probability of first implantation was 0.8% and 4% for replacement of the device [3, 17].

There were several hypotheses for the source and mechanism of CIED infection. One was contamination of the pocket tissue before the implanting operation. After implantation, patients' defense capabilities shifted and previously dormant bacteria massively propagated, leading to infection [18]. In total, 6.7% patients with positive bacteria were infected by the same bacteria that were also the same as the bacteria isolated from the device [10]. Under this hypothesis, asymptomatic bacterial colonization led to the infection after the device-replacing operation, which explained why the infection rate of the second implantation was higher than that of the first. Bacterial contamination was a new problem in operations and a tough problem with more and more patients receiving ICD treatment, especially combined with cardiac resynchronization therapy devices. Bacterial contamination was related to the operating time and the number of implanted devices. The ICD patients who suffered from serious heart trouble were easier to be infected, which would affect the analysis for cost and effectiveness in ICD patients' survival time [34]. In addition, remnants of CIED* in vivo* might increase the risk of infection. Complete removal of all CIED hardware should be attempted at the time of upgrade and revision and even prior to orthotopic heart transplantation [35, 36]. Furthermore, gene polymorphisms, such as fibronectin-binding protein A of* S. aureus*, were associated with infection of cardiovascular devices [37].

## 5. Limitations

Although bacterial DNA determination had high sensitivity, the possibility of a few false-negative results must be considered. In addition, false-positives could not be completely excluded, which might be affected by factors of the implantable device, antibiotic therapeutic regimen, the* in vivo* location of the implanting pocket, and contamination. The number of monocentric samples was limited; therefore some important issues, such as whether asymptomatic bacterial colonization on CIEDs could lead to clinical infection, could not be answered. Although quantitative PCR was used, it was impossible to quantify the bacteria completely on and around the CIED, which might be an important factor for pathogenicity. Recent research had indicated that means of sonic degradation were conducive to bacterial determination after replacement, removal, and infection [38–40].

## 6. Conclusions

There was a high proportion of asymptomatic bacteria on pacemakers or in ICD patients. The determination rate of coagulase-negative* Staphylococcus* was the highest. The major carried bacteria were related to common microflora in CIED infection, and bacteria rarely resulting in CIED infection were detected. The functions of these bacteria in CIED infection, for example, synergism, facilitating form of biofilm, or protection, needed further research.

## Figures and Tables

**Table 1:** DNA results of 78 patients.

Specimen	Positive number (*n*)	Percentage (%)
Overall	30	38.5
Pocket tissue	18	23.1
Surface of device	23	29.5
Both specimens	11	14.1

**Table 2:** Bacterial species determined by DNA technology (*n*, %).

Species	Positive number (*n*)	Percentage (%)
*Staphylococcus aureus *	1	3.3
*Coagulase-negative Staphylococcus *	9	30.0
*S. epidermis *	4	13.3
*S. saprophyticus *	2	6.7
*S. warneri *	2	6.7
*S. hominis *	1	3.3
*Streptococcus viridans *	2	6.7
*Pseudomonas aeruginosa *	3	10.0
***Propionibacterium acnes***	3	10.0
*Corynebacterium parvum *	4	13.3
*Klebsiella pneumoniae *	2	6.7
*Enterobacter cloacae *	3	10.0
*Escherichia coli *	4	13.3
***Acinetobacter baumannii***	1	3.3

**Table 3:** Baseline characteristics of 78 patients.

	DNA positive (*n* = 30)	DNA negative (*n* = 48)	χ^2^	*P*
Age	69.1 ± 15.2	67.8 ± 17.1	1.0838	0.2819
Gender			0.624	0.429
Male	17	23		
Female	13	25		
PM indications			0.707	0.702
SSS	14	20		
AVB	10	21		
AF with long intervals	6	7		
Replacement time			Fisher	0.004
1 time	22	46		
2 times	6	2		
3 times	2	0		
PM types			Fisher	1.0
Single chamber	10	15		
Double chamber	19	31		
ICD/CRT	1	2		
Temporary PM application	3	2	Fisher	0.381

PM, pacemaker; SSS, sick sinus syndrome; AVB, atrioventricular block; AF, atrial fibrillation with long intervals.

**Table 4:** Physiological characteristics of 78 patients.

	DNA positive (*n* = 30)	DNA negative (*n* = 48)	*t*	*P*
Body mass index	25.22 ± 4.78	26.02 ± 4.02	0.7974	0.4277
White blood cells (×10^9^/L)	6.23 ± 2.1	6.06 ± 2.2	0.34	0.7348
Blood platelets (×10^9^/L)	230.24 ± 40.21	226.98 ± 35.78	0.3748	0.7088
Hemoglobin (g/L)	135.46 ± 8.94	136.59 ± 8.08	0.5793	0.5641
Total serum protein (g/L)	63.66 ± 6.58	64.28 ± 6.69	0.4031	0.688
Serum albumin (g/L)	42.64 ± 4.03	44.05 ± 4.12	1.4919	0.1399
Ejection fraction (EF %)	54.68 ± 8.76	56.69 ± 7.49	1.0838	0.2819

**Table 5:** Comorbidities and drug application.

	DNA positive (*n* = 30)	DNA negative (*n* = 48)	χ^2^	*P*
Chronic heart disease	12	14	0.669	0.413
Coronary heart disease	8	10	0.216	0.642
Hypertension	14	22	0.02	0.886
Atrial fibrillation			Fisher	0.631
Paroxysmal	6	5		
Persistent	3	6		
Permanent	3	5		
Dilated cardiomyopathy	6	6	Fisher	0.526
Diabetes	7	8	0.372	0.542
Renal insufficiency	6	2	Fisher	0.053
Chronic systolic HF	6	11		0.672
COPD	2	3	Fisher	1.0
Immunosuppressor	1	2	Fisher	1.0
Warfarin	1	2	Fisher	1.0
Antiplatelet drug use	20	15	8.026	0.005
Antibiotic use	11	3	10.741	0.001
Malignancy	1	1	Fisher	1.0
Bacterial infection history	29	11	36.787	0.0001
Surgical history	3	4	Fisher	1.0

HF, heart failure; COPD, chronic obstructive pulmonary disease.

**Table 6:** Multifactor logistic regression analysis.

Risk factor	*β*	SE (*β*)	Wald	*P*	OR	95% CI
Bacterial infection history	3.596	0.793	21.79	<0.0001	36.45	6.85–198.231
Antibiotic use	2.104	0.695	7.934	0.004	8.198	2.01–30.47
Antiplatelet drug use	1.324	0.474	7.362	0.005	3.758	1.287–9.645
Replacement of two times	1.732	0.818	4,126	0.031	5.65	1.131–30.82
Renal insufficiency	1.634	0.831	3.871	0.041	5.12	1.004–26.73

Replacement times: significant difference between 2 and 1 times, no difference between 3 and 1 times.
